# Collecting Real-Time Patient-Reported Outcome Data During Latent Labor: Feasibility Study of the MyCap Mobile App in Prospective Person-Centered Research

**DOI:** 10.2196/59155

**Published:** 2024-11-08

**Authors:** Katherine Kissler, Julia C Phillippi, Elise Erickson, Leah Holmes, Ellen Tilden

**Affiliations:** 1 College of Nursing Anschutz Medical Campus University of Colorado Aurora, CO United States; 2 School of Nursing Vanderbilt University Nashville, TN United States; 3 College of Nursing University of Arizona Tucson, AZ United States; 4 School of Nursing Oregon Health & Sciences University Portland, OR United States; 5 School of Medicine Oregon Health & Sciences University Portland, OR United States

**Keywords:** patient-reported outcomes, survey methods, smartphone, labor onset, prodromal symptoms, prospective studies

## Abstract

**Background:**

The growing emphasis on patient experience in medical research has increased the focus on patient-reported outcomes and symptom measures. However, patient-reported outcomes data are subject to recall bias, limiting reliability. Patient-reported data are most valid when reported by patients in real time; however, this type of data is difficult to collect from patients experiencing acute health events such as labor. Mobile technologies such as the MyCap app, integrated with the REDCap (Research Electronic Data Capture) platform, have emerged as tools for collecting patient-generated health data in real time offering potential improvements in data quality and relevance.

**Objective:**

This study aimed to evaluate the feasibility of using MyCap for real-time, patient-reported data collection during latent labor. The objective was to assess the usability of MyCap in characterizing patient experiences during this acute health event and to identify any challenges in data collection that could inform future research.

**Methods:**

In this descriptive cohort study, we quantified and characterized data collected prospectively through MyCap and the extent to which participants engaged with the app as a research tool for collecting patient-reported data in real time. Longitudinal quantitative and qualitative surveys were sent to (N=18) enrolled patients with term pregnancies planning vaginal birth at Oregon Health Sciences University. Participants were trained in app use prenatally. Then participants were invited to initiate the research survey on their personal smartphone via MyCap when they experienced labor symptoms and were asked to return to MyCap every 3 hours to provide additional longitudinal symptom data.

**Results:**

Out of 18 enrolled participants, 17 completed the study. During latent labor, 13 (76.5%) participants (all those who labored at home and two-thirds of those who were induced) recorded at least 1 symptom report during latent labor. A total of 191 quantitative symptom reports (mean of 10 per participant) were recorded. The most commonly reported symptoms were fatigue, contractions, and pain, with nausea and diarrhea being less frequent but more intense. Four participants recorded qualitative data during labor and 14 responded to qualitative prompts in the postpartum period. The study demonstrated that MyCap could effectively capture real-time patient-reported data during latent labor, although qualitative data collection during active symptoms was less robust.

**Conclusions:**

MyCap is a feasible tool for collecting prospective data on patient-reported symptoms during latent labor. Participants engaged actively with quantitative symptom reporting, though qualitative data collection was more challenging. The use of MyCap appears to reduce recall bias and facilitate more accurate data collection for patient-reported symptoms during acute health events outside of health care settings. Future research should explore strategies to enhance qualitative data collection and assess the tool’s usability across more diverse populations and disease states.

## Introduction

Recognition of the importance of the patient experience has increased focus on patient-reported outcomes and symptom measures in medical research [1-3]. However, patient-reported data are susceptible to bias that limits reliability [4]. Prospective patient-reported data collection is difficult because symptom onset can be unpredictable and often begins outside of a health care setting. Indeed, most research describing patient symptom experiences relies on retrospective patient-reported data collection, which is highly susceptible to recall bias [4]. To improve validity, researchers have recently implemented mobile technologies to collect patient-generated health data in real time [5-7]. Most implementations of mobile symptom reports have surveyed patients on a daily or weekly basis and have focused on chronic conditions, especially cancer [5,6,8]. Reported benefits of mobile collection of patient-generated symptom data include increased data quality, facilitation of shared decision-making, and improved symptom management [9]. Extending the technology to include patients with acute health events with more frequent symptom reporting could provide important research to guide clinical practice.

To meet the growing needs of researchers to collect patient-generated health data, the REDCap (Research Electronic Data Capture) consortium developed MyCap, a smartphone app specifically designed for mobile collection of patient-generated health data [10-12]. Unlike stand-alone apps, MyCap integrates with REDCap allowing for seamless use by REDCap’s 3.2 million users worldwide [10,13]. REDCap is a secure web-based software platform (compliant with HIPAA [Health Insurance Portability and Accountability Act]) designed to support data capture for research studies, providing (1) an intuitive interface for validated data capture, (2) audit trails for tracking data manipulation and exports, (3) automated export procedures for seamless data downloads to common statistical packages, and (4) procedures for data integration and interoperability with external sources [10-12]. Like REDCap, MyCap is a free, easy-to-use, and research-regulation compliant interface for smartphone use. Multiple options are available for sending surveys including scheduled push-outs, participant-initiation, and manual invitations that can be repeated for longitudinal data collection [10,14].

As a use case for the collection of patient-reported symptom data using MyCap during an acute health event in the community setting, we evaluated the feasibility of MyCap for research data collection during latent labor. Latent labor, the time between first labor symptoms and active labor (approximately 6 cm cervical dilatation) [15-18], is a critical period when patients rely on symptoms to determine when to present to a birthing facility. Presenting for care either too early or too late may put the maternal/infant dyad at risk [15,19-22]. However, the onset and expected duration of latent labor have historically been difficult to define, predict, and characterize, and the current research guiding practice relies primarily on retrospective patient surveys. Like other acute health events, latent labor symptoms onset is difficult to predict, often occurs at home, and symptom evaluation is critical to shared decision-making and timely delivery of health care [7,14,23-29]. The purpose of this study was to evaluate the feasibility of MyCap in characterizing patient experiences of latent labor symptoms as a use case for MyCap as a tool for prospective collection of patient-generated research data during an acute health event. We sought to identify and address potential issues in data collection to guide future use in prospective, patient-reported research.

## Methods

### Recruitment and Consent

In this observational cohort feasibility study, patients were recruited and enrolled at the end of pregnancy in the outpatient setting and followed through approximately 6 weeks postpartum. Self-reported symptoms and experiential data were collected via MyCap as part of a larger study characterizing the relationship between biomarkers associated with labor onset and progression.

Low-risk nulliparous individuals (those experiencing their first labor) are the populations identified by national organizations for targeted cesarean prevention efforts [15,30]. Thus, we recruited nulliparous individuals with term pregnancies and a single fetus in the vertex position planning vaginal birth from outpatient prenatal clinics at Oregon Health Sciences University from June 2021 through January 2023. Oregon Health Sciences University Center for Women’s Health clinics provide comprehensive care including prenatal care through clinics staffed by certified nurse midwives, obstetric residents, and faculty obstetricians in an academic medical center. The clinic also facilitates perinatal research through a dedicated research team.

Patients were screened using chart review and approached at clinic visits, via phone, or by email through the electronic medical record at approximately 37 weeks estimated gestational age if they met inclusion criteria including nulliparity, maternal age of 20-45 years, viable, term pregnancy (≥ 37 weeks gestation), able to understand English and provide informed consent, and access to a smartphone with cell or internet service. Exclusion criteria were multiparty, multiple gestation, pre-existing complex comorbidities (eg, pre-eclampsia), documented fetal congenital anomaly, preterm birth, persistent breech fetal position at term, fetal death prior to onset of labor, and indication for planned cesarean birth. Participants who expressed initial interest in the study were sent the consent form to review and invited to meet with the research team to review the protocol and discuss questions.

### Ethical Considerations

This study was approved by the Oregon Health Sciences University institutional review board (#0020328). All participants completed a full informed consent process and provided written consent for study participation and secondary analysis of data. Study data were deidentified and securely stored to protect human subject privacy and confidentiality in compliance with HIPAA regulations. Participants were compensated with a US $25 gift card for the first study visit and a US $125 gift card following completion of the final study visit.

### Data Collection in MyCap

Following consent to participate, participants completed a demographic survey (age, marital status, zip code, race/ethnicity, educational completion, sexual orientation, 3 questions characterizing childbirth education, and three 6-point Likert-like scale questions about preparedness for childbirth).

Entering the participant’s information into REDCap during enrollment automatically generated an individual QR code within the MyCap system that the participant used to open the app. Once the app was opened, study participants were guided through a short orientation and completed a practice version of the MyCap symptom survey in-person with a study team member. MyCap was used to collect patient-reported quantitative and qualitative data on labor symptoms experienced during the end of pregnancy through the transition to active labor.

**Figure 1:**
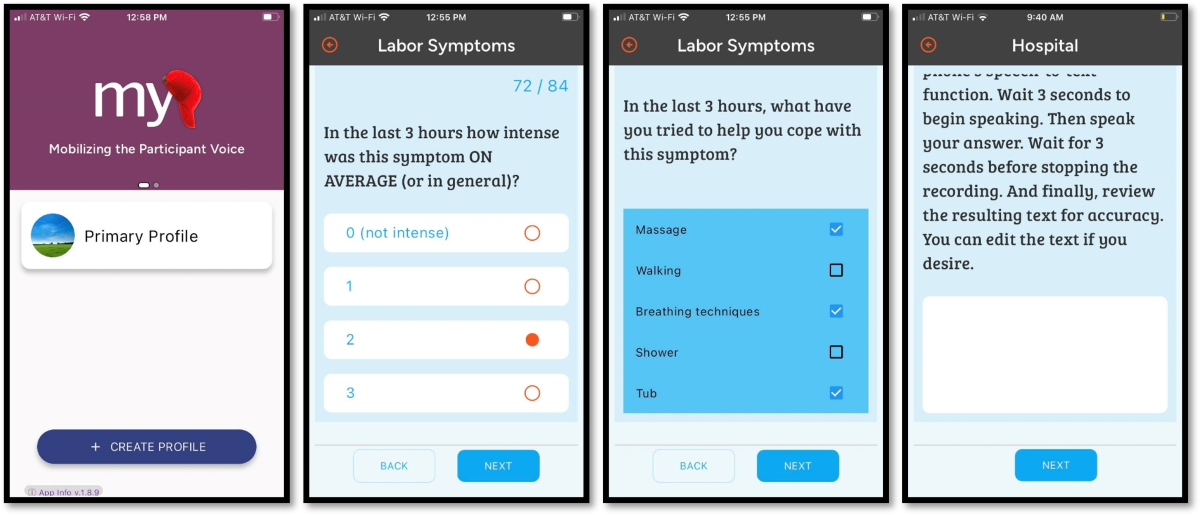
Screenshots of the labor symptom survey administered to nulliparous pregnant patients via the MyCap App used to evaluate feasibility of collecting symptom experience data in real-time during early labor. Four screenshots demonstrate how different elements of the MyCap app appear on the participant’s smartphone. Using PROMIS (Patient-Reported Outcomes Measurement Information System) measures, participants were asked about symptoms, symptom intensity/duration, and coping. Participants could also provide responses to open-ended questions using text or speech-to-text technology.

Participants were invited to initiate the MyCap survey with their personal smartphone when they experienced symptoms they perceived to be the onset of labor (Figure 1). The survey consisted of 10-point Likert-like scale, childbirth-specific, symptom-specific PROMIS (Patient-Reported Outcomes Measurement Information System) measures to quantify and characterize labor symptoms (eg, intensity, frequency, duration, and coping), and open-ended questions to identify relevant qualitative themes and context [29]. Once the survey was initiated, MyCap sent a survey reminder via text message every 3 hours until the participant indicated that symptoms had stopped or progressed to active labor. If symptoms stopped, participants were able to pause data collection until symptoms resumed. Data entered into MyCap was directly transmitted and stored electronically in a REDCap database.

As symptoms progressed, participants were given the opportunity to provide qualitative data about the symptom experience either by typing or using a voice-to-text option. The voice-to-text tool included in MyCap allowed participants to record responses and then review and revise the transcript before submitting. Qualitative prompts included identification of what was surprising about the experience of latent labor and how participants made the decision to present to the birthing facility. Two weeks after birth, participants were alerted to complete a final survey via MyCap, text, or email (per patient preference) that captured information on labor onset and latent labor including support persons, the decision to present in labor, and factors contributing to hospital admission.

### Analysis

We enrolled a sample size (N=18) sufficient to provide feasibility data on the proportion of participants that complete MyCap surveys, the number of submissions on symptom data in latent labor per participant, and how participants engage in open-ended questions. This sample size was also expected to adequately provide data to contextualize findings and compare MyCap engagement between individuals with spontaneous versus induced labor.

Primary outcomes included the number of participants who engaged in MyCap during latent labor, the number of quantitative and qualitative surveys participants submitted, the number and types of symptoms participants reported, and analysis of the quality of qualitative responses. Descriptive statistics were used to quantify and characterize the data that were collected via MyCap. Proportions were used to describe the number of participants who engaged and the extent to which they used different MyCap features. Secondarily, qualitative data were analyzed using thematic analysis by the primary author who has training in advanced qualitative methods [31-33].

## Results

### Demographics and Labor/Birth Outcomes

A total of 18 participants enrolled; 1 participant withdrew prior to labor onset (N=17). Most participants identified as White (13/17), had at least a college degree (13/17), and were married (13/17; Table 1). Participants were evenly divided between preferring to be contacted by email versus text message. No participants preferred phone or voicemail. Half of the participants had engaged in childbirth education.

Five (29%) participants started labor spontaneously and 12 (71%) participants were induced. Most participants had a vaginal birth (14/17, 82%) and 3 (18%) participants had a cesarean birth. Nine (59%) participants received oxytocin during their labor while 7 (41%) participants labored without oxytocin.

**Table 1:** Demographics of participants in this prospective feasibility study of low-risk nulliparous patients reporting early labor symptoms via the MyCap app.

Demographics			Values
**Race/Ethnicity, n (%)**			
	Non-Hispanic White	13 (81.3)	
	American Indian/Pacific Islander	1 (6.3)	
	Hispanic White (Mexican)	1 (6.3)	
	Mixed race (Non-Hispanic Black/White)	1 (6.3)	
Age (years), mean (SD)			29.81 (21.75-36.25)
**Marital status, n (%)**			
	Single	0 (0)	
	Partnered	3 (18.8)	
	Married	13 (81.3)	
**Educational completion, n (%)**			
	< High school	1 (6.3)	
	High school	0 (0)	
	Some college but did not complete college degree	2 (12.5)	
	College	6 (37)	
	Master degree or equivalent	2 (12.5)	
	PhD or equivalent	5 (31.3)	
**Household income (US $), n (%)**			
	<25,000	1 (6.3)	
	25,000-50,0000	2 (12.5)	
	50,000-75,000	1 (6.3)	
	75,000-100,000	1 (6.3)	
	100,000-125,000	1 (6.3)	
	125,000-150,000	6 (37.5)	
	150,000-175,000	1 (6.3)	
	175,000-200,000	3 (18.8)	
	>200,000	0 (0)	
**Preferred contact method (n=15)**			
	Text	7	
	Email	7	
	Text or email	2	
	Voicemail	0	
Childbirth education class, n (%)			8 (50)
**Childbirth education type, n (%)**			
	Lamaze	1 (12.5)	
	Through OHSU	5 (62.5)	
	Other	2 (25.0)	

### Quantity and Quality of MyCap Data

During latent labor, 13 participants engaged MyCap and recorded at least 1 symptom report. There were a total of 191 quantitative symptom reports entered in real-time during latent labor (mean of 10 per participant) (Table 2). Four participants provided real-time qualitative survey responses during latent labor with a total of 6 voice-to-text or written responses. Qualitative responses during labor tended to be brief, typically 2-3 words long.

In the postpartum period, 14 participants responded to additional qualitative prompts. These responses tended to be longer and provide context for the latent labor symptom reports and short qualitative responses entered in labor.

**Table 2:** Quantity of data reported on the MyCap app from low-risk nulliparous pregnant participants during the prospective cohort study evaluating the feasibility of app use to collect real-time patient-reported data during early labor.

Data type	Quantity
Symptom reports	13 with at least 1 symptom report Total of 191 symptom reports (avg of 10/participant)
Qualitative Survey Responses: Voice-to-test or Written	5 participants (6 responses during labor) 15 participants (15 responses postpartum)

### Symptoms and Coping

Fatigue was the most frequently reported symptom (12/13 participants reported feeling tired and 9/13 feeling very tired; Table 3). Contractions and pain were reported by 11/13 participants. Nausea was reported in just 2 participants, but had the highest rating of intensity (mean maximum intensity 6.50), the most bothersome to participants (mean of 5.00), and had the lowest coping score (mean of 8.50). Diarrhea was similarly uncommon (4/13 participants), but also bothersome (mean of 4.33). Anxiety was common (7/13 participants reported feeling anxious) and was associated with lower coping scores (mean of 7.94). Many participants also reported feeling excited (8/13 participants). Three participants also noted other physical symptoms including: rectal pain, bladder pain, cramping, acid reflux, loss of mucus plug, dizziness, and insomnia. Seven participants reported other emotional symptoms including feeling: emotional, upset, frustrated, and calm.

**Table 3:** Frequency, intensity, and coping for labor symptoms reported by low-risk nulliparous participants in this prospective cohort study.

Symptom	Number of participants who reported symptom	Total number of symptom reports	Average maximum intensity (0-10)	Mean general intensity (0-10)	Average score of how bothersome a symptom was (0-10)	Average coping score (0-10)
Sample	13	N/A^a^	N/A^a^	N/A^a^	N/A^a^	N/A^a^
Contractions	11	76	3.09	2.42	2.41	8.62
Pain	11	79	4.23	3.30	3.37	8.54
Nausea	2	3	6.50	4.50	5.00	8.50
Diarrhea	4	12	5.25	5.08	4.33	9.00
Leaking water	3	11	N/A^b^	N/A^b^	N/A^b^	8.73
Leaking blood	3	8	N/A^b^	N/A^b^	N/A^b^	9.63
Headache	3	5	N/A^b^	N/A^b^	N/A^b^	9.20
Fatigue/tired	12	71	N/A^b^	N/A^b^	N/A^b^	8.69
Anxiety	7	16	N/A^b^	N/A^b^	N/A^b^	7.94
Excitement	8	16	N/A^b^	N/A^b^	N/A^b^	N/A^b^

^a^N/A: not applicable.

^b^Intensity, how bothersome a symptom was, and the coping score was evaluated for PROMIS (Patient-Reported Outcomes Measurement Information System) measure symptoms. Presence of symptoms was identified as common pregnancy symptoms requiring further medical evaluation and emotional experiences.

### Admission Characteristics

Of the 13 participants who reported latent labor symptoms, the duration of symptoms prior to hospital admission averaged 21.72 (range 6-68) hours. Eight participants reported calling care providers (6 spoke with a midwife, 2 spoke with a nurse) at least once prior to admission and 5 were advised to stay home longer. Of participants with spontaneous labor onset, 2 were admitted in active labor (≥6 cm cervical dilation), 2 were admitted at 4-5 cm dilation, and 1 was admitted in latent labor (≤3 cm dilation).

### Qualitative Responses

Four participants provided qualitative survey responses during labor responding to questions about what was surprising and how they decided when to present to the birthing facility. Themes from responses included desiring more anticipatory guidance about the intensity and pain of contractions and wanting more specific information about when to present to the birthing facility.

…intensity of pain, blood, 3-1-1

participant 3

Most participants (14/17) completed the postpartum survey (all via MyCap) and 8 included qualitative responses. Participants completed the survey at a mean of 18 (range 1-41) days postpartum. Qualitative responses recorded postpartum were longer and more detailed than qualitative responses recorded during labor. Many of the postpartum qualitative responses related to the experience of telephone triage prior to admission to the hospital in labor. Themes included feeling reassured by the provider to continue laboring at home, confused by conflicting instructions on contraction frequency (ranging from every 2-5 minutes apart), and unsure about how to gauge contraction intensity.

Helpful to have a calming, knowledgeable resource on the other end of the line. Helped me feel more comfortable staying and laboring at home and less worried about not coming in right away. Helped me understand what was going on with my body and what to expect

participant 13

At first I didn't feel like I was being listened to when I told them I thought I was in labor and that it felt different from prodromal labor.

participant 10

## Discussion

### Principal Findings

Most participants engaged actively with the MyCap app to report symptoms experienced during latent labor demonstrating feasibility in this use case for prospectively collecting patient-reported data from patients with acute health events. We found that participants in our study reported more symptoms than described in previous literature characterizing latent labor symptoms. For example, in our feasibility sample, a third of engaged participants reported nausea or diarrhea compared with only 3.5%-6.9% in a cohort study by Petersen et al [34] of (N=549) nulliparas and (N=490) multiparas describing symptoms reported during hospital admission for labor [27]. Similarly, over half of the participants in our study who reported symptoms stated that they had emotional symptoms (anxiety and excitement), whereas only 2.7%-3.4% had emotional symptoms in the Petersen et al cohort study [34]. Our findings suggest that participant reporting in real-time may increase the accuracy of reporting and be helpful for understanding how patients experience unfolding health conditions in real time. This is consistent with the literature on ecological momentary assessment which posits that measuring behavioral and cognitive processes at the time they occur and in the normal ecological setting of participants usually increases the validity of the measure [35,36].

All participants who started labor spontaneously at home engaged with the MyCap app during latent labor and two-thirds of the induced participants engaged with the app as well. We expect that engagement with MyCap during latent labor mimics the use of commercial apps for recording symptoms (eg, contractions) during labor, the use of which has increased dramatically over the last 10 years [37,38]. Patients engage apps for labor including contraction timing, suggestions for support, and symptom tracking; efforts are underway to advance the evidence for the use of apps that provide responsive recommendations for early labor support [37-39].

Engagement with qualitative survey questions in MyCap was limited during labor, which likely reflects a preference for engagement with quantitative rather than qualitative survey questions during acute symptom events. More participants provided qualitative reports retrospectively about labor symptoms. Using both mixed methods collected both prospectively and retrospectively may optimize the accuracy and depth of data.

Addressing bias in patient-reported outcome measures is a priority to ensure the accuracy of research and clinical evaluation [4,40]. We used MyCap to mitigate bias by (1) collecting data in real-time to reduce recall bias, (2) sending survey reminders to participant’s personal phones to reduce nonresponse bias [41], (3) obtaining data directly from participants to reduce proxy response bias, and (4) contextualizing real-time data with retrospective surveys postpartum to address timing bias. Additional considerations to address threats to validity when using MyCap include: using validated surveys and tools for the population of interest and piloting surveys among the population of interest with specific attention to factors that contribute to fatigue bias (length of surveys, frequency of surveys in longitudinal studies, and burden of surveys on participants) [4].

### Strengths and Limitations

This feasibility study successfully leveraged the novel MyCap technology to gather real-time data on patient experiences during latent labor in the community setting, for which little to no published data are available. However, some limitations are acknowledged to inform future study design.

Inferential statistics and generalizability are limited by the small sample size. Instead, we were able to provide descriptive analysis. In addition, the findings were limited by low response rates and brief or thin responses on qualitative surveys completed during labor, likely because of pain and progressing labor that demanded participant’s attention. Qualitative responses were elaborated in responses to the later postpartum survey.

Because the surveys were initiated by the participants, the amount of missing data is unknown. It was impossible to differentiate periods when participants were not experiencing labor symptoms from periods when participants had symptoms but did not enter them. For example, periods of heavy symptom burden may preclude participants from engaging in the app.

Additionally, the sample included primarily White, English-speaking, and college-educated people. This study required in-person consent and MyCap training and recruitment coincided with the COVID-19 pandemic which significantly hindered and may have biased recruitment. We hope that MyCap can be used to improve the understanding of experiences of all birthing people, especially to amplify the voices of marginalized populations to address health disparities and improve safety and experience of labor care [14]. Future research is needed to evaluate MyCap in diverse patient populations and to collect user experience data to inform continued app development.

MyCap is focused on data entry, which limits functionality that would potentially benefit patients or encourage engagement. Additional tools such as personalized recommendations, interactive reports, or symptom-tracking feedback would provide participants with immediate value and feedback from the data they submit and balance the one-sidedness of data provision.

### Future Considerations

In addition to demonstrating the feasibility of MyCap as a research tool, the following lessons can be learned to support effective research design. Patient-reported symptom data can be strengthened by periodically sending surveys to all participants (eg, daily) to better characterize whether participants deny symptoms prior to when they initiate the survey. Future surveys of latent labor symptoms should include those additional symptoms that participants described (physical: rectal pain, bladder pain, cramping, acid reflux, loss of mucus plug, dizziness, and insomnia; emotional: feeling emotional, upset, frustrated, and calm).

Surveys that are not responded to should be re-sent to participants. Patients experiencing a significant health event, including laboring or postpartum participants, may need additional opportunities to complete surveys. Adding retrospective follow-up interviews or asking participants to contextualize or respond to the data they entered during latent labor may add richness and depth to surveys completed in real time.

### Conclusions

This preliminary study demonstrates the feasibility of use of MyCap as a research tool to prospectively collect symptom data in the community setting during latent labor to accurately facilitate timely care. Interestingly, we found that people were more likely to engage with the app at home than while they were in the hospital, although integration with inpatient research teams might encourage more participation. Participants were also more likely to engage in quantitative survey questions than qualitative ones during labor. The retrospective qualitative data were helpful in contextualizing the quantitative findings and may be an opportunity for triangulation of data through mixed methods research.

While we used the tool to collect data on latent labor, MyCap may be useful for other health conditions or transitions that unfold outside of health care settings. This real-time, patient-led tool will support longitudinal data collection from participants in future research and provide a critical opportunity for a better understanding of unfolding health conditions.

## Abbreviations

HIPAA: Health Insurance Portability and Accountability Act
PROMIS: Patient-Reported Outcomes Measurement Information System
REDCap: Research Electronic Data Capture

## Data Availability

The datasets generated or analyzed during this study are not publicly available, but are available from the corresponding author on reasonable request.
